# De-escalation of empiric antibiotic therapy in sepsis - an indian observational study

**DOI:** 10.1186/2197-425X-3-S1-A405

**Published:** 2015-10-01

**Authors:** S Jakkinaboina, K Swarna Deepak

**Affiliations:** Apollo Health City, Critical Care Medicine, Hyderabad, India

## Introduction

The guidelines for an appropriate use of antibiotic therapy recommend de-escalation as soon as the culture sensitivity results are available.There was a great need to study the frequency of de-escalation, no change and escalation of antibiotics and its impact in septic patients in an Indian ICU setting in view of rising burden of drug resistance and health care cost.

## Objectives

To determine the rate of de-escalation of the empiric antimicrobial treatment in ICU patients admitted with sepsis. The characteristics of antimicrobial treatment associated with de-escalation & its impact on ICU length of stay, hospital length of stay (LOS) and 30 day mortality were determined.

## Methods

We carried out a prospective observational study enrolling patients admitted to the ICU with sepsis from 01/02/2014 to 01/03/2015. Both the medical and surgical units of the ICU were included. **"No change"** was defined as when empiric therapy was maintained without modification, **"Escalation of therapy"** was defined when there’s a switch to or addition of an antibiotic with a broader spectrum, and **"De-escalation"** when there’s a switch to or interruption of a drug class resulting in a less broad spectrum of coverage. If antimicrobial change consisted of escalation and de-escalation (i.e. switch to or addition of an antibiotic with a broader spectrum but also withdrawal of another antibiotic), the patient was assigned to "escalation group" for statistical analysis.

## Results

The total number of patients enrolled were 315 out of which 17 patients were excluded due to death before culture results were available. The characteristics observed among the therapeutic strategy groups were seen in tables 1, 2.

**Table 1:** Patient characteristics with therapeutic strategy.

Variable	No change n	De-escalation n	Escalation n	P value
Number of patients	148	74	76	
Age in years	57.62	57.81	58.18	0.96
HOSPITAL length of stay in days	11.41	10.68	19.21	0.0001
APACHE II score at admission	15.3	15.84	20.11	0.0001
SOFA score at admission	6.07	6.65	8.08	0.004
Number of patients expired at 30 days after admission	20	10	26	0.0001
number of medical patients	144	64	66	0.003
Number of patients requiring vasopressors	20	16	34	0.0001
ICU length of stay in days	5.85	6.24	12.42	0.0001

**Table 2:** Patient characteristics with therapeutic strategy.

Variable	No change n	De-escalation n	Escalation n	P value
Number of initial empirical antibiotics 1	58	26	32	0.007
Number of initial empirical antibiotics 2	72	26	36	
Number of initial empirical antibiotics 3	18	22	8	
Most Common initial empirical antibiotic	beta lactam +beta lactamase inhibitor	Carbapenem	beta lactam +beta lactamase inhibitor	0.0001
Most Common Escalated /De-escalated Antibiotic	beta lactam +beta lactamase inhibitor	beta lactam +beta lactamase inhibitor	Carbapenem followed by colistin	0.0001
Multi drug resistant organisms	6	8	34	0.0001
ESBL organisms	4	4	10	0.0001
Most Common Source of infection	Lung	Lung	Lung	
H1N1 POSITIVE patients	29	10	6	0.062

The most common antibiotic after de-escalation& antibiotic in No Change group was Betalactam + beta lactamase inhibitor.

The statistically significant factors which increased the 30 day mortality are Escalation of antibiotics, Increased ICU LOS, Increased APACHE II and SOFA scores at admission, medical patients, vasopressor requirement, lung as the source of infection, Infection with MDR organisms.

The statistically significant factors which increased the ICU LOS are use of vasopressors, Comorbidities, APACHE II at admission, SOFA at admission, MDR organisms, escalation of antibiotic.

## Conclusions

Escalation of antibiotics resulted in statistically significant increase in the mortality and increased length of stay in the ICU and hospital. No significant differences were observed with respect to ICU length of stay, Hospital length of stay and 30 day mortality among No Change & De Escalation groups.

**Figure 1:**
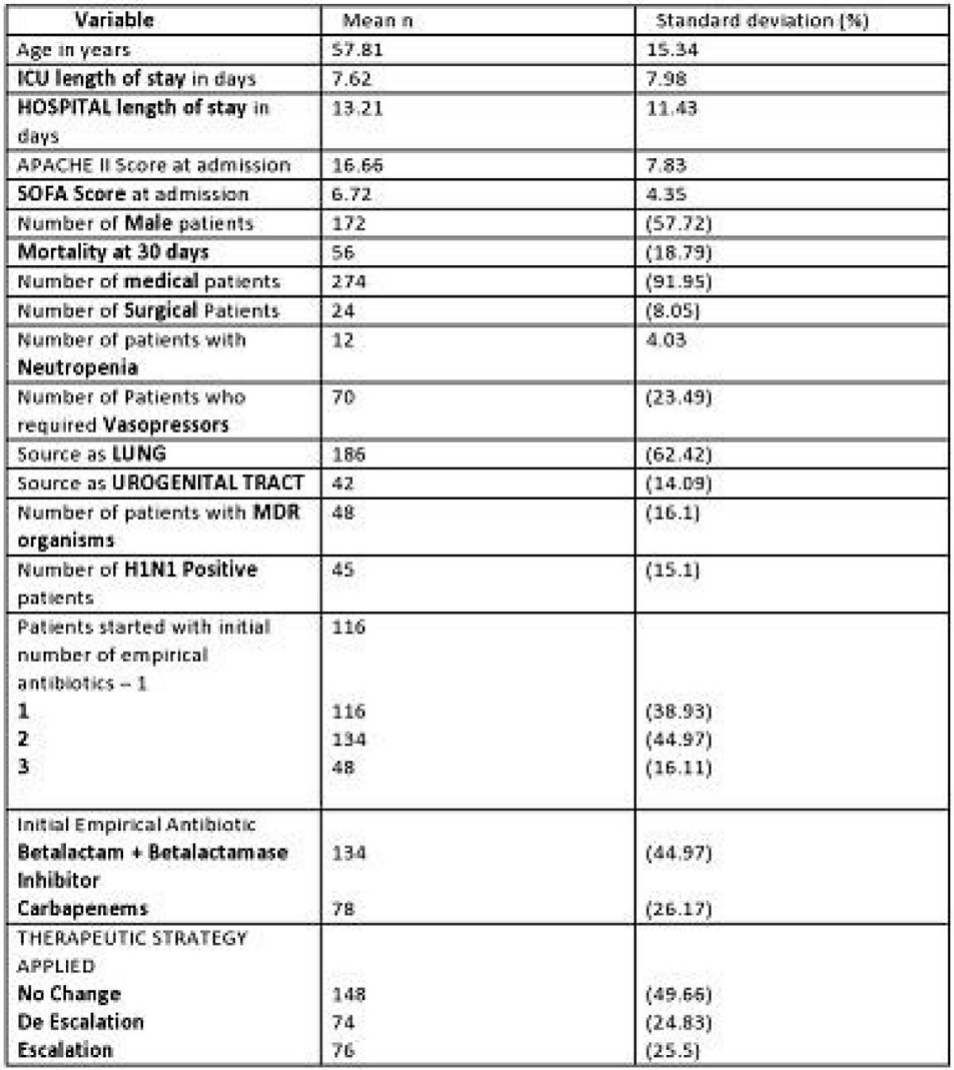
**The characteristics of the overall group of patients.**

**Figure 2:**
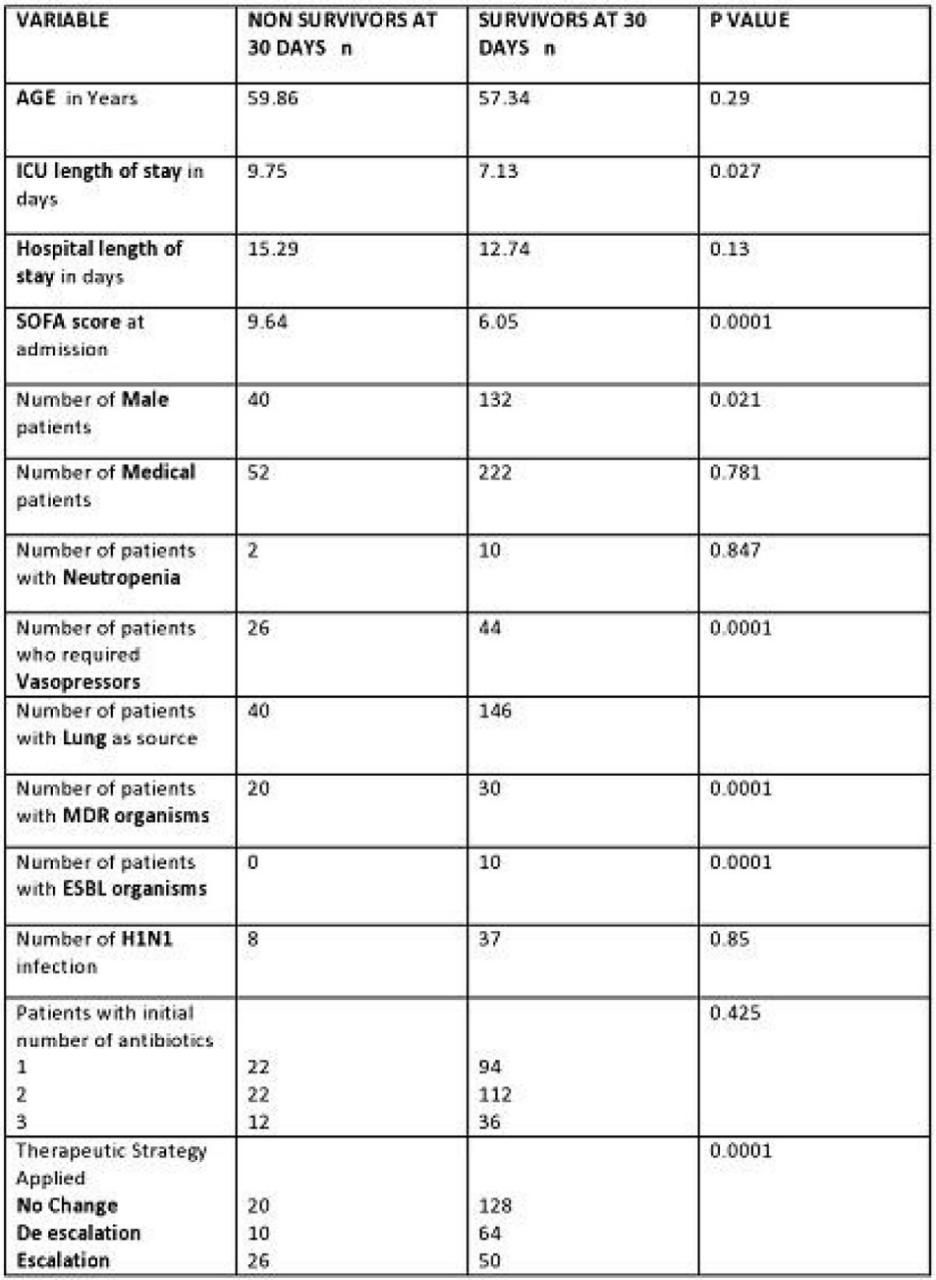
**Comparison between survivors and non survivors at 30 days.**

**Table 3:** Prevalence of organisms in patient groups. The frequency of organisms in culture positive cases.

ORGANISM ISOLATED	NO CHANGE n	DE ESCALATION n	ESCALATION n	Total n (%)
Fungal infections	4	2	8	14(9.39%)
Non ESBL Gram Negative organisms	12	10	8	30(20.13%)
ESBL Gram negative organisms	4	4	10	18(12.08%)
MDR organisms	6	8	34	48(32.21%)
MRSA organisms	0	6	0	6(4.02%)
MSSA organisms	0	2	2	4(2.68%)
Vancomycin Resistant Enterococcus	2	0	0	2(1.34%)
Stenotrophomonas maltophila	4	4	4	12(8.05%)
